# Multiple early local transmissions of chikungunya virus, Mainland France, from May 2025

**DOI:** 10.2807/1560-7917.ES.2025.30.32.2500545

**Published:** 2025-08-14

**Authors:** Lucie Fournier, Guillaume André Durand, Amandine Cochet, Elise Brottet, Caroline Fiet, Quiterie Mano, Catarina Krug, Laura Verdurme, Thomas Blanchot, Rémi Fournier, Investigation team, Marie-Claire Paty, Gilda Grard, Florian Franke, Clémentine Calba, Investigation team, Delphine Casamatta, Anne Guinard, Nadège Marguerite, Gisèle Gay

**Affiliations:** 1 Santé publique France (French National Public Health Agency), Saint Maurice, France; 2 National Reference Center for Arboviruses, National Institute of Health and Medical Research (Inserm) and French Armed Forces Biomedical Research Institute (IRBA), Marseille, France; 3 Unité des Virus Émergents (UVE: Aix-Marseille University-University of Corsica-French National Research Institute for Sustainable Development (IRD) 190-National Institute of Health and Medical Research (Inserm) 1207-French Armed Forces Biomedical Research Institute (IRBA)), Marseille, France; 4 Santé publique France (French National Public Health Agency), Montpellier, France; 5 Santé publique France (French National Public Health Agency), Lyon, France; 6 Santé publique France (French National Public Health Agency), Strasbourg, France; 7 Santé publique France (French National Public Health Agency), Marseille, France; 8 Laboratoire Cerba, Frépillon, France; 9 Laboratoire Eurofins Biomnis, Lyon, France; 10 Laboratoire Inovie, Montpellier, France; 11 The members of the Investigation team are listed under Collaborators at the end of the article

**Keywords:** chikungunya, arbovirus, France, autochthonous transmission, aedes albopictus

## Abstract

In 2025, a large outbreak of chikungunya occurred in Réunion Island (France). By 10 July, 1,911 imported cases were notified in Mainland France, most (89%) were associated with travel to Réunion Island. Ten autochthonous outbreaks in five French regions involving 27 cases were identified between May and mid-July and outbreaks occurred earlier than in previous years. *Aedes albopictus,* the mosquito vector, is endemic in large parts of France. Transmission events early in the vector active season is concerning.

Réunion Island (France), a French overseas region, experienced a major chikungunya outbreak in 2025, involving a viral strain well adapted to *Aedes albopictus,* the mosquito vector present in Mainland France [1]. From 1 January to 30 April 2025, 1,291 imported chikungunya cases were notified in Mainland France, the highest number since the surveillance began in 2006. Here we describe autochthonous transmission events of chikungunya virus (CHIKV) in Mainland France, early in the *Ae. albopictus* active season.

## Surveillance of *Aedes*-borne viruses in Mainland France

Chikungunya, dengue and Zika are mandatorily notifiable diseases in France. Between May and November, the months when the vector is active, arbovirus surveillance is enhanced to prevent or limit autochthonous transmission. During this enhanced surveillance, public health authorities aim to improve case detection through awareness raising campaigns towards healthcare professionals and daily monitoring of laboratory results. To lower the risk of autochthonous transmission, all cases are investigated to ascertain the place of infection, of viraemia and to implement control measures around imported and autochthonous cases. Autochthonous cases are thoroughly investigated by door-to-door investigations to find other cases. Control measures to contain an outbreak are: (i) vector control measures, like identification and suppression of vector breeding sites and treatment of the areas of transmission with insecticides and larvicides; (ii) recommendations to viraemic people to reduce bite risks by trying to stay at home while being viraemic and using mosquito sprays; (iii) screening blood donations from the area. In addition to the above-mentioned control measures, local healthcare professionals are contacted, and local communities are informed by placing posters or flyers in pharmacies, waiting rooms and via municipal websites.

## Imported chikungunya cases

**Figure 1:**
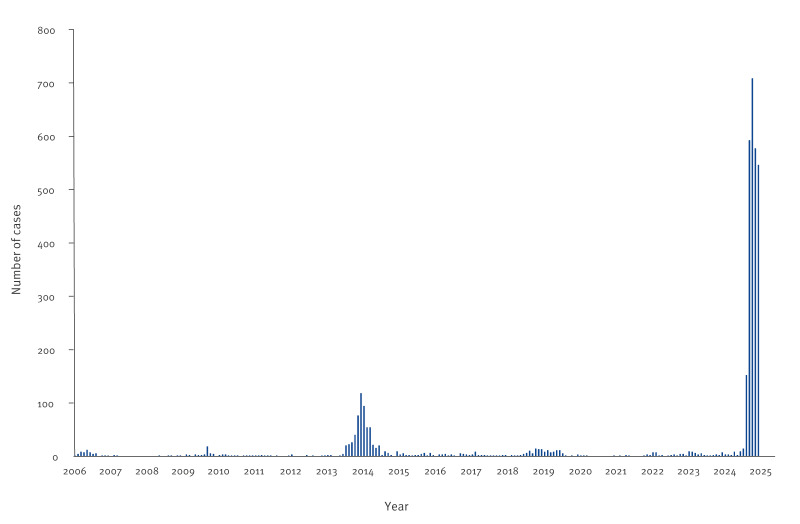
Number of imported cases of chikungunya, Mainland France, 2006–10 July 2025 (n = 3,671)

Symptom onset was reported from 620 imported chikungunya cases between May and June 2025 (Figure 1). Most (n = 484; 78%) had travelled to Réunion Island. More than 86% (533/620) of the imported chikungunya cases were viraemic in Mainland France, and 91% (485/533) of the viraemic cases resided in departments colonised by the vector.

## Autochthonous transmission of chikungunya virus

By 10 July 2025, 10 autochthonous CHIKV transmission events comprising 27 cases were identified in five regions of Mainland France (Table). The number of cases per event was low (≤ 3) except for one event with 13 cases.

**Table:** Autochthonous transmission events of chikungunya virus, Mainland France, May–10 July 2025 (n = 10)

Place of transmission			Number of autochthonous cases	Date of symptom onset		Likely primary imported case identified (place of travel, if known)
Municipality	Department	Region		Earliest	Latest	
Prades-le-Lez	Hérault	Occitanie	1	27 May	27 May	No
La Crau	Var	Provence-Alpes-Côte d'Azur	2	2 Jun	5 Jun	Yes (Réunion Island)
Salon-de-Provence	Bouches-du-Rhône	Provence-Alpes-Côte d'Azur	13	16 Jun	30 Jun	No
Grosseto-Prugna	Corse-du-Sud	Corse	3	19 Jun	27 Jun	No
Montoison	Drôme	Auvergne-Rhône-Alpes	3	13 Jun	19 Jun	Yes (Réunion Island)
Bernis	Gard	Occitanie	1	11 Jun	11 Jun	Yes (Réunion Island)
Lipsheim	Bas-Rhin	Grand-Est	1	26 Jun	26 Jun	No
Claix	Isère	Auvergne-Rhône-Alpes	1	1 Jul	1 Jul	Yes (Réunion Island)
Fréjus	Var	Provence-Alpes-Côte d'Azur	1	1 Jul	1 Jul	No
Castries	Hérault	Occitanie	1	2 Jul	2 Jul	No

**Figure 2:**
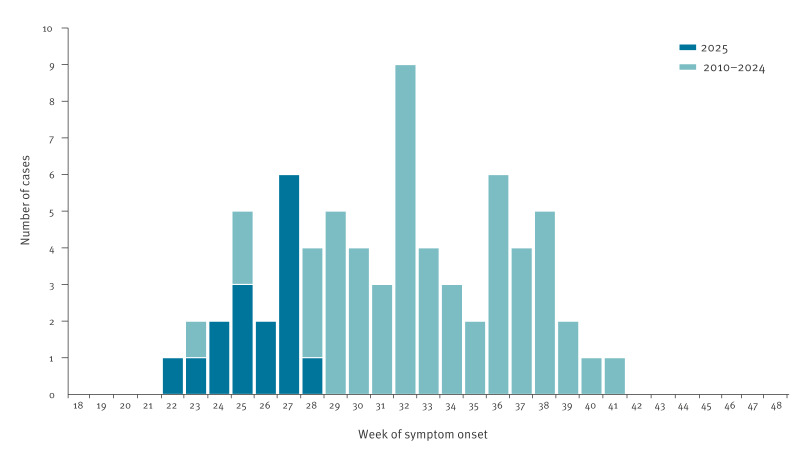
Week of symptom onset for the first autochthonous cases of dengue virus, chikungunya virus and Zika virus of a local transmission event, Mainland France, 2010–10 July 2025 (n = 53)^a^

^a^ The first case of an autochthonous event is included in the number of cases.

For the first time in Mainland France, an autochthonous CHIKV transmission event occurred in May, and six autochthonous transmission events started in June (Figure 2). Prior to 2025, the earliest date recorded for onset of symptoms of an autochthonous case of arbovirus infection in Mainland France was 12 June, and only three of the 53 previously documented local transmission events had started in June. In earlier years, autochthonous arbovirus transmission occurred later with 12 of 53 of autochthonous events starting in July, 22 of 53 in August and 16 of 53 in September.

Door-to-door surveys were carried out around autochthonous cases to search for the primary case and for other autochthonous cases in nine local transmission events. In four events, investigations led to the identification of the likely primary case (Table), all of them had travelled to Réunion Island.

The median reporting delay for imported chikungunya cases with an onset date after 1 May 2025 was 9 days after symptom onset (interquartile range (IQR): 6–16 days), which is relatively short.

**Figure 3:**
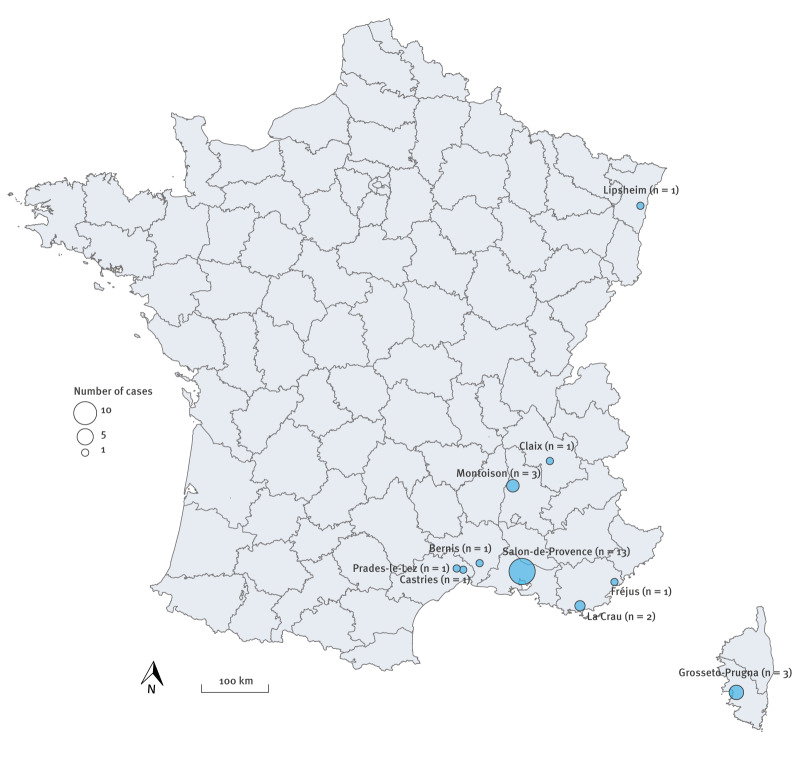
Map of the autochthonous transmission events of chikungunya virus, Mainland France, May–10 July 2025 (n = 27)

Nine autochthonous transmission events occurred in regions where *Ae. albopictus* has been established at least over a decade (13–21 years) and where autochthonous arbovirus transmission by *Ae. albopictus* has been previously documented (Provence-Alpes-Côte d’Azur, Occitanie, Corse and Auvergne-Rhône-Alpes). However, autochthonous transmission was detected for the first time in the Grand Est region (Figure 3).

## Discussion

The risk of local transmission of *Aedes-*borne viruses such as dengue, chikungunya and Zika viruses in Mainland France increases with the number of imported cases and the growing spread of *Ae. albopictus* across the territory. In previous years, the number of imported cases has correlated with the epidemiological situation outside of Mainland France, especially the French outermost regions (French Guiana, Guadeloupe, Martinique, Mayotte, Réunion Island and Saint-Martin), which are also part of the European Union. The large chikungunya outbreak in Réunion Island in 2025 raised concerns of autochthonous transmission in Mainland France via imported cases. In response, healthcare professionals were informed of the risk of transmission during the vector active period and a press briefing on the subject was released at the beginning of the high-risk period (May).

Since 2017, local transmission events of arboviruses have been identified annually with a considerable increase since 2022 [2-4]. In 2025, the autochthonous chikungunya cases occurred very early in the *Ae. albopictus* activity season, when vector densities are still expected to be low [5,6]. This could be partly explained by both the high number of imported cases and the CHIKV strain being well adapted to *Ae. albopictus* [1].

For the first time, we documented transmission in the Grand Est region. In the Bas-Rhin department (Grand Est), where the transmission occurred, *Ae. albopictus* was detected in 2015 and > 60% of the inhabitants live in municipalities where the presence of this vector species has been documented. The occurrence of autochthonous transmission of CHIKV in a department with a relatively recent vector colonisation is concerning and highlights the risk faced even in the northern parts of France, and elsewhere in Europe, where *Ae. albopictus* is progressively expanding to new areas [7].

So far, most autochthonous transmission events have been small, showing the effectiveness of the surveillance in quickly identifying autochthonous transmission and of the response system in containing the transmissions. The cases were identified soon after the symptom onset, and measures to prevent further transmission were promptly enacted. However, the investigations and measures taken around autochthonous cases are very resource intensive, and the surveillance and response systems are not scaled for such a high number of autochthonous transmission events. If the current trend in autochthonous transmission were to continue, this system would be put under considerable stress and might find itself overwhelmed [8].

Although we were able to detect small autochthonous transmission events, there might be undetected autochthonous transmission of CHIKV that could result in wide-scale dissemination.

In previous studies on the determinants of autochthonous transmission of dengue virus and CHIKV in Mainland France, delayed reporting of imported cases to local public health authorities was the main driver of autochthonous transmission [9]. If the number of autochthonous cases increases, investigations of imported cases may be deprioritised, leading to delayed investigations and implementation of control measures for those cases. This may in turn increase the risk of autochthonous transmission.

## Conclusion

The occurrence of multiple local transmissions of CHIKV in Mainland France, early in the 2025 season, illustrates the increasing threat posed by arboviral diseases in France and Europe. These events could put under stress public health institutions, health systems and vector control resources. Policy makers should anticipate this increasing risk by allocating appropriate resources, developing adequate contingency plans and promoting research.

## Data Availability

Data on autochthonous transmission of chikungunya, dengue and Zika are available on the website of Santé publique France and updated weekly from May to November.
